# How to cut child mortality in half again: making primary healthcare work

**DOI:** 10.7189/jogh.16.03010

**Published:** 2026-04-17

**Authors:** Susanne Carai, Sophie Jullien

**Affiliations:** 1 World Health Organization Regional Office for Europe, Division of Country Health Policies and Systems, Child and Adolescent Health, Copenhagen, Denmark; 2 Witten/Herdecke University, Global Child Health, Witten, Germany

## Abstract

The gap between what primary healthcare (PHC) could achieve and what it currently delivers remains wide. Preventable child deaths remain a reality, particularly in underserved communities, and hard-won gains risk being reversed by diminishing attention. Making PHC work for children and adolescents requires clear, evidence-based standards, supportive health systems, and community-driven engagement. The WHO Pocket Book of Primary Health Care for Children and Adolescents and its mobile application bridge these pillars into one actionable strategy. Over the past decades, global child mortality has fallen by nearly 60%, largely due to services delivered through PHC. Yet millions of preventable deaths persist, reflecting weaknesses in governance, financing, workforce, and quality of care. Selective interventions contributed to earlier progress but narrowed the Alma Ata vision, limiting PHC to a small set of conditions. The Pocket Book and its app provide an integrated, evidence-based framework spanning birth to 18 years that operationalises comprehensive PHC. Country adaptation processes act as catalysts for systems change by identifying and correcting health systems’ dysfunctions and engaging parents and caregivers as partners in care. This approach offers a practical pathway to halving child mortality again.

## PRIMARY HEALTHCARE: A VISION STILL TO BE REALISED

The gap between what primary healthcare (PHC) could achieve and what it currently delivers remains wide – but is bridgeable. Estimates suggest that PHC can deliver more than 90% of all health interventions essential to achieving universal health coverage [1]. Health systems with stronger PHC are associated with better population health and greater equity, while containing costs [2]. Investments in PHC have also been shown to improve equity and access, healthcare performance, accountability of health systems, and health outcomes [3]. Strategic levers to enable PHC implementation have been identified in the World Health Organization (WHO)’s PHC Operational Framework, namely: political commitment and leadership; governance and policy frameworks; funding and resource allocation, and engagement of communities and other stakeholders [4].

The virtues of PHC have been sung for many decades, at least since the declaration of Alma-Ata in 1978. Vision and evidence are clear and a compelling economic case for PHC has been made repeatedly, yet governments around the world continue to underfund these services, and the quality of care at PHC level remains substandard, with a lack of concrete leadership, training, and tools [1,5,6].

## FROM SELECTIVE INTERVENTIONS TO COMPREHENSIVE PHC: REDESIGNING CHILD HEALTH

Selective strategies such as the Growth monitoring, Oral rehydration therapy, Breastfeeding and Immunization (GOBI) and the Integrated Management of Childhood Illness (IMCI) scaled up life-saving interventions for children and are being credited – at least in part – for halving child mortality in recent decades [7,8]. However, they also narrowed the original vision of Alma Ata by restricting PHC to a limited set of diseases and conditions [9]. In 2018, WHO and the United Nations Children’s Fund initiated a process to revisit and redesign global child health approaches [10]. This process concluded that attaining the Sustainable Development Goals (SDGs) required a substantial shift in thinking about child and adolescent health in order to reduce childhood mortality and improve quality of care for children [11]. The focus needed to move beyond the survival of children under five years of age towards a more holistic view of child and adolescent health, with greater attention to health promotion, disease prevention, early risk factor management and monitoring of chronic conditions [12]. Following disruption during the COVID-19 pandemic, this effort culminated in 2022 with the release of the WHO Pocket Book of Primary Health Care for Children and Adolescents (hereafter referred to as the Pocket Book), expanding guidance across the full age range from birth to 18 years [13]. This resource integrates promotive, preventive, diagnostic, and curative guidance, moving beyond selective programmes towards comprehensive, continuous care.

## STRENGTHENING PHC BY BRIDGING ITS MAIN PILLARS: THE ROLE OF THE POCKET BOOK AND COUNTRY-LEVEL ADAPTATION

**Figure 1:**
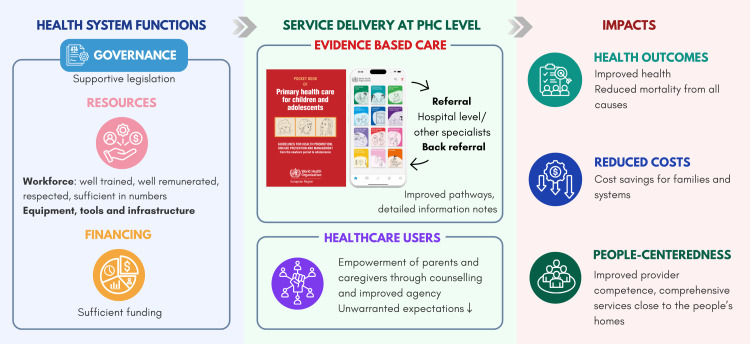
The WHO Pocket Book of Primary Health Care for Children and Adolescents and its app bridge health systems and community pillars into one actionable strategy.

The Pocket Book and its mobile application support PHC providers in delivering evidence-based care in the best interests of children and adolescents. Country adaptation processes are led by national technical working groups – typically convened by ministries of health or professional associations – which bring together primary care providers, referral level clinicians, public health experts, and academic and standard-setting institutions. In this process, countries are not passive recipients, but co-developers, contextualising recommendations, aligning them with national policies, and ensuring feasibility within existing service delivery structures. Importantly, adaptation is not limited to tailoring the Pocket Book to existing policies: it also involves critically reviewing national guidelines and policies to assess whether they enable the delivery of evidence-based care and, where needed, adapting policies and systems to support implementation. Adaptation processes, therefore, act as a catalyst for systems change by concretely identifying and correcting dysfunctions in governance, including weaknesses in referral pathways, resources (workforce, medicines, and equipment), and financing. The implementation of the Pocket Book and its app engage parents and caregivers as empowered partners in care, reinforcing shared responsibility for health outcomes. The Pocket Book and app offer a concrete and practical vehicle for realizing the potential of PHC by bridging health systems and community pillars into one actionable strategy (**Figure 1**).

### Standards and evidence-based quality care

The Pocket Book sets evidence-based standards for the services every child and adolescent should be able to access at primary healthcare level. It operationalises competence – defined as the ability to correctly diagnose and manage illness according to evidence-based standards – by providing symptom- and complaint-based algorithms. The app also includes a weight-based dosing calculator to reduce medication errors and decision support tools to curb inappropriate antibiotic use and strengthen management of non-communicable diseases. The Pocket Book and app include content on health promotion and disease prevention, particularly through well-child visits, supporting a proactive approach to child and adolescent health.

Evidence shows that the competence of healthcare providers is consistently ranked by people as the most valued health system attribute [14]. It is thus not only a professional requirement, but also a population expectation.

The mobile application format enables easy point-of-care access for healthcare providers and allows for easier updates than printed materials, addressing long-standing barriers to guideline uptake in routine practice. Its user-friendly design further enhances usability in busy clinical settings.

### The country adaptation and implementation process

In the three years following its publication, over 20 countries – primarily through the WHO Regional Office for Europe and Pan American Health Organization – have requested translation and adaptation of the Pocket Book, underscoring the growing global demand for practical, context-sensitive guidance to strengthen primary healthcare at the frontline [15–23].

### Correcting health system (dys-)functions

Translation and country-specific adaptation of the Pocket Book are essential to ensure uptake and implementation by local PHC providers, yet technical guidance alone is insufficient for ensuring quality of care and improving child and adolescent health outcomes [24]. To achieve impact, national adaptation must go beyond translation and adaptation processes must be explicitly linked to the core functions of health systems.

#### Governance

Good governance is essential for turning the standards of the Pocket Book from guidance on paper into services delivered in practice. The adaptation process can help with identifying and correcting governance dysfunctions. For example, PHC providers can only implement services outlined in the Pocket Book if governance ensures the necessary conditions are in place: adequate equipment and infrastructure, uninterrupted supply of essential paediatric consumables and medicines outlined in the Pocket Book, the inclusion of Pocket Book standards in publicly financed insurance schemes or government benefit packages. Payment mechanisms must also recognise the time required for preventive services, counselling, and longitudinal follow-up. While the Pocket Book and its adaptation processes cannot resolve political instability, conflict, or corruption, they can still play two critical roles. First, the national adaptation process creates a structured space for ministries of health and partners to explicitly surface and discuss governance constraints – including financing gaps, regulatory barriers, and system bottlenecks – rather than leaving them implicit. Second, the Pocket Book provides a practical, standardised clinical tool that frontline providers can continue to use even amid instability, leadership turnover, or system disruption, which helps maintain continuity and quality of care even in fragile settings.

Legislative reforms are part of the governance responsibility. They are needed to enable providers to apply the standards in practice – for example, allowing adolescents to receive care according to their maturity, even without parental consent in some circumstances. In many contexts, restrictive laws prevent adolescents from accessing confidential health services [25,26]. Similarly, regulations that mandate hospitalisation for certain conditions result in unnecessary admissions and prevent evidence-based care from being delivered at the PHC level [27–29].

Governance is also critical for referral systems. The Pocket Book defines which services can be safely delivered at the PHC level and when to refer. In many settings, referral is currently practiced in a vacuum: patients are told to go elsewhere without clear instructions or consideration of feasibility, often entering a ‘black box’ and returning without information for the PHC provider on how to continue and coordinate care for the child or adolescent. To fulfil their coordinating role, PHC providers need information from specialist and referral level providers. Clear referral standards need to be mandated and implemented, thereby ensuring bidirectional communication between PHC and hospitals or specialist services, overcoming the long-standing animosity between specialists and primary care providers.

There are successful examples of how countries have leveraged the adaptation process to improve governance. One country in Central Asia is reviewing its legislation on mandatory screenings, using the Pocket Book as reference, to phase out non-evidence-based practices [16]. In another, the adaptation process created an opportunity to identify and address regulatory gaps concerning the minimum requirements needed to ensure quality home visits [22,23].

#### Workforce

A well-trained, well-remunerated workforce sufficient in numbers is central to delivering high-quality ambulatory care for children and adolescents. Pre-service curricula for medicine and nursing must be revised to include comprehensive training in this area. The Pocket Book provides a foundation for such reform, offering clear standards that can be embedded into curricula and used as a basis for examination and certification. Promising examples already exist. One country has integrated the Pocket Book into the curricula of several universities and adopted it as a standard reference for examination and certification, while another is currently in the process of integration [23,30].

Training innovations, including modular online learning, simulation, and game-based education, are under development to accelerate capacity building at scale [31].

Ensuring adequate remuneration and recognition for PHC providers who care for children and adolescents is critical. In many countries, PHC providers remain underpaid and remuneration gaps between countries are extremely wide [24,25]. Increased competencies and wider responsibilities must be matched with professional status and fair pay.

#### Financing

All the above reforms require adequate and sustained financing. Evidence consistently shows that investing in PHC leads to cost reduction in the long term through improved outcomes, reduced hospital admissions, and efficient use of resources [2,3,32]. Financing arrangements must therefore cover health services defined in the Pocket Book free of charge at the point of care for children and their families, including essential medicines and supplies, fair remuneration for providers and adequate support for preventive and longitudinal services, recognising PHC as cost-effective investment rather than a budgetary burden. Across diverse health system contexts, this can be operationalised through pragmatic mechanisms such as rebalancing spending towards PHC within existing budgets, embedding Pocket Book services into publicly financed benefit packages, aligning provider payment mechanisms to incentivise prevention and continuity of care, and protecting PHC budgets from short-term political cycles. In many countries, financing barriers are less about absolute resource scarcity and more about competing priorities and hospital-centric spending patterns [32].

### Healthcare users: caregiver and community empowerment and managing unwarranted expectations

The Pocket Book contributes to caregiver and family empowerment by promoting shared understanding of evidence-based care practices, reinforcing their role as full partners in care. Structured counselling on breastfeeding, responsive caregiving, nutrition, home care of common childhood illnesses, and other health concerns from birth through adolescence enhances agency and supports informed decision-making. Children and adolescents are likewise empowered to take part in decisions affecting their health and well-being, in line with their evolving capacities and the United Nations Convention on the Rights of the Child [33]. At the community level, engagement is needed to strengthen understanding that watchful waiting is not substandard care but, in some cases, the best clinical decision, demonstrating that doing ‘nothing’ can sometimes mean doing the most. This includes addressing common expectations, such as the perceived need for antibiotics to treat viral infections like the common cold, where such treatment is not clinically indicated. The counselling boxes model respectful, caregiver-centred communication, including, for example, evidence-based management of acute otitis media in situations where antibiotics are not indicated, and support providers in explaining watchful waiting with clear guidance on when and where to seek further care if symptoms worsen or fail to improve. Countries that have completed adaptations have so far largely retained the original content, suggesting that the core counselling principles resonate across diverse cultural settings.

Building trust in the health system and its workforce is essential to support informed decision-making, promote appropriate care-seeking behaviours, and foster shared responsibility for health outcomes.

## HALVING CHILD MORTALITY AGAIN: THE NEXT FRONTIER FOR PHC

Over the past three decades, substantial progress has been made in reducing global child mortality, in large part thanks to services delivered at the PHC level [7,8]. The total number of under-five deaths worldwide has declined from 12.8 million in 1990 to 4.8 million in 2023 [34]. Moreover, the global under-five mortality rate has dropped by 59% in the last three decades, from 93 deaths per 1000 live births in 1990 to 37 in 2023 [34,35]. While these achievements represent one of global health’s major successes, preventable deaths remain a reality, particularly in underserved communities, and hard-won gains risk being reversed by diminishing attention. Latest estimates show an increase in 2024, to 4.9 million children dieing before reaching their fifth birthday, with an additional 2.1 million deaths among older children, adolescents and youth aged 5–24 years [36].

Achieving another halving of child mortality will require not only sustaining existing interventions, but also transforming how health systems organise and deliver PHC for children and adolescents. Competing financing priorities, workforce constraints, governance challenges, and the political economy underpinning them remain major risks to achieving this transformation.

The potential gains of this agenda are considerable. Comprehensive PHC can further reduce mortality from pneumonia, diarrhoea, malaria, and neonatal complications, while also improving management of common chronic conditions such as asthma, autism and diabetes [1,32].

Expanded adolescent services can strengthen mental health support and reduce unintended pregnancies. Promotion and prevention efforts are critical to reducing the burden of non-communicable diseases beginning in childhood and extending into adulthood, as well as preventing injuries and exposure to violence [13,32].

Health systems can increase efficiency by reducing avoidable hospitalisations and inappropriate medicine use, while families benefit from reduced catastrophic expenditures and access to care closer to home. Early identification of diseases allows timely and often less costly treatment, improving outcomes, increasing efficiency, and ultimately saving lives [13,32].

## CONCLUSIONS

Cutting child mortality in half again is an achievable goal – but not through vertical campaigns, selective interventions, technological fixes, or abstract notions of PHC. It requires aligning evidence-based clinical guidance with supportive health system functions: governance, financing, workforce, medicines and equipment, and community engagement and responsibility. The Pocket Book and its app provide a practical framework for comprehensive, evidence-based care for children and adolescents, while national adaptation processes offer an opportunity to identify and correct underlying health system dysfunctions. It also supports the empowerment of children and adolescents, families and communities by promoting shared understanding of appropriate care practices, fostering trust in the health workforce, and encouraging active participation in health decision.

The WHO Pocket Book of Primary Health Care for Children and Adolescents and its country adaptation processes can bridge the pillars needed for successful health service delivery into one coherent and actionable strategy, turning the long-recognised vision of PHC into real-world impact for children, adolescents, and their families.
